# A Novel Framework for Anomaly Detection for Satellite Momentum Wheel Based on Optimized SVM and Huffman-Multi-Scale Entropy

**DOI:** 10.3390/e23081062

**Published:** 2021-08-17

**Authors:** Yuqing Li, Mingjia Lei, Pengpeng Liu, Rixin Wang, Minqiang Xu

**Affiliations:** 1 Deep Space Exploration Research Center, Harbin Institute of Technology, Harbin 150080, China; bradley@hit.edu.cn (Y.L.); wangrx@hit.edu.cn (R.W.); xumq@hit.edu.cn (M.X.); 2 Naval Research Academy, Beijing 100061, China; newtime1987@163.com

**Keywords:** satellite momentum wheel, anomaly detection, Huffman-multi-scale entropy (HMSE), support vector machine (SVM), adaptive particle swarm optimization (APSO)

## Abstract

The health status of the momentum wheel is vital for a satellite. Recently, research on anomaly detection for satellites has become more and more extensive. Previous research mostly required simulation models for key components. However, the physical models are difficult to construct, and the simulation data does not match the telemetry data in engineering applications. To overcome the above problem, this paper proposes a new anomaly detection framework based on real telemetry data. First, the time-domain and frequency-domain features of the preprocessed telemetry signal are calculated, and the effective features are selected through evaluation. Second, a new Huffman-multi-scale entropy (HMSE) system is proposed, which can effectively improve the discrimination between different data types. Third, this paper adopts a multi-class SVM model based on the directed acyclic graph (DAG) principle and proposes an improved adaptive particle swarm optimization (APSO) method to train the SVM model. The proposed method is applied to anomaly detection for satellite momentum wheel voltage telemetry data. The recognition accuracy and detection rate of the method proposed in this paper can reach 99.60% and 99.87%. Compared with other methods, the proposed method can effectively improve the recognition accuracy and detection rate, and it can also effectively reduce the false alarm rate and the missed alarm rate.

## 1. Introduction

As important spacecraft, study of the reliability of artificial satellites is a hot topic at present. Generally, an artificial satellite consists of a structural system, temperature control system, attitude control system, measurement and control system, and power supply system. The mission of the attitude control system is to help the satellite achieve attitude stability or attitude maneuver, to further guarantee the normal operation of the satellite platform and the normal work of the payload.

Satellites have high requirements for attitude accuracy, which makes the task of attitude control systems very heavy. Health state and reliability are the basic guarantee for the normal operation of satellites [1]. Therefore, research on the theory and technology of automatic fault diagnosis and anomaly detection of satellite attitude control systems will further ensure the safe and reliable operation of on-orbit aircraft, reducing the possibility of space accidents.

In recent years, many scholars have conducted research on fault diagnosis technology or health management technology. These research contents can be roughly divided into three main aspects. First, when there is a specific research object, a feasible solution is to construct a simulation model of the object by analyzing the working mechanism and failure mode of the object. The data generated based on the simulation data is used as the theoretical prediction value, and then the judgment criterion is designed to complete the detection task. Luo et al. propose an improved phenomenological model based on meshing vibration to generate fault simulation data [2]. Li et al. established an INS/ADS fault detection model based on kinematic equations, and combined an unscented Kalman filter (UKF) with Runge-Kutta to deal with the non-linear and discretization problem [3]. Second, some research aims at extracting the fault features by constructing more effective signal processing methods, such as the feature extraction method based on entropy value [4,5], the feature extraction method based on spectral kurtosis time (Spectral Kurtosis, SK) [6], or the Frequency domain feature extraction method [7]. To fully excavate the features of the momentum wheel telemetry signal, this paper uses a combination of time domain features, frequency domain features and complexity features for feature extraction. Considering that, compared with permutation, dispersion, hierarchy, etc., sample entropy has better consistency for different parameters, this paper chooses a complex quantification method based on sample entropy. Third, for the fault recognition process, various pattern recognition methods are used to learn the mapping relationship between features and failure modes, so as to realize automatic fault recognition [8].

Due to the extremely complex structure and working principle of the spacecraft itself, and the strong coupling between the sub-systems, it is very difficult to construct an accurate simulation model of the spacecraft or its components [9,10]. As the spacecraft is affected by the special space environment during its orbiting operation, it is extremely prone to unpredictable failures, for example, the circuit signal disturbance caused by electromagnetic background [11], the sudden change of attitude caused by the impact of space debris [12], etc. In addition, during the process of the spacecraft downloading telemetry data to the ground-based measurement and control station, data jumps and even partial loss can occur [13]. Therefore, the data generated by the simulation model is often difficult to simulate the actual telemetry data of the spacecraft, and it becomes very difficult to use the spacecraft anomaly detection method based on the physical simulation model in practical applications.

The fault diagnosis method based on the data mode does not impose necessary restrictions on the prior knowledge of the object or system (including mathematical models and expert experience, etc.), such as artificial neural network (ANN), support vector machine (SVM), Bayesian network (BN) and other health assessment methods.

ANN is a method that is widely used in fault identification problems. Multilayer Perceptron (MLP) is the most typical type of feedforward neural network model, which usually uses a BP algorithm to learn the parameters of the model. Kumar et al. proposed a method based on principal component analysis (PCA) and MLP to detect and classify the three-phase current signals online [14]. In addition, probabilistic neural network (PNN) [15], RBF neural network [16], extension neural network (ENN) [17] and recurrent neural network (RNN) [18,19,20] have also been applied to fault detection and diagnosis problems.

For high-dimensional identification problems in fault diagnosis, the SVM method based on the principle of structural risk minimization has been widely used in recent years [21,22,23]. Compared with the ANN method based on the principle of empirical risk minimization, the learning goal of the SVM is to learn the optimal classification hyperplane in the feature space. The ANN has the ability to deal with pattern recognition problems, but the sample size is large, and it takes a long time to adjust the network structure parameters. Bayesian decision-making has significant execution ability under the premise of considering prior probability, but good accuracy is based on a prior model with appropriate assumptions. Compared with the above methods, the SVM only needs a small number of samples for training and has better generalization ability. Therefore, this paper chooses SVM as the means of pattern recognition. In the field of fault diagnosis, research on the SVM method mainly focuses on two aspects of obtaining more accurate recognition accuracy, i.e., by optimizing the hyperparameters of the model and constructing a new kernel function. For specific recognition tasks, to optimize the hyperparameters of the model to obtain better recognition performance, many optimization methods are applied [24,25,26]. Liu et al. proposed a novel small sample data missing filling method based on support vector regression (SVR) and genetic algorithm (GA) to improve the equipment health diagnosis effect [25]. Particle swarm optimization (PSO) is a hyperparameter optimization algorithm which is used by Cuong-Le et al. for damage identifications [26]. In terms of constructing a new kernel function, Wang et al. proposed a kernel function selection mechanism under sparse representation and the superiority of the selection mechanism was performed in simulations and engineering experiments involving high-speed bearing fault diagnosis [27]. Although both GA and PSO can solve high-dimensional complex optimization problems well, in the iterative process of PSO, the particles can retain the memory of the good solution, but the GA cannot, so PSO can often converge to a better solution more quickly. Based on the above analysis, this paper uses PSO to optimize the multi-class SVM.

From the above analysis, it can be seen that the following problems still exist in the direct application of existing anomaly detection or fault diagnosis methods to the anomaly detection problem of the satellite momentum wheel.

(1) Due to the complex structure and control law of the satellite momentum wheel itself, it is very difficult to construct an accurate simulation model, so model-based anomaly detection methods often fail to achieve satellite momentum wheel anomaly detection.
(2) Satellite telemetry data often contains outliers (due to the data with very large deviations introduced by the telemetry process). These data alone cannot characterize the health of the spacecraft, but they can easily be detected as abnormal values by existing methods. At the same time, some segments of the telemetry data are lost in the process of downloading the data from the satellite to the ground. Therefore, reasonable preprocessing of telemetry data is required.
(3) The sampling frequency of telemetry data collected by on-orbit satellite is often less than 1Hz, and the data itself has a long change period, so traditional anomaly detection methods based on time-frequency domain analysis are difficult to work with telemetry data.

Therefore, in response to the above problems, this article proposes a new method based on multi-type features fusion and improved SVM to handle the problem of anomaly detection for the satellite. The main contributions of the proposed framework can be summarized as follows:

(1) We design a new anomaly detection framework for satellites, which includes a telemetry data preprocessing part, a telemetry data multi-type feature extraction part, and a data-driven anomaly detection part.
(2) We propose a new method to construct the fusion-feature sequence HMSE-T/F. The HMSE-T/F is based on the Huffman-multi-scale entropy and the selected time/frequency-domain feature. The Huffman-multi-scale entropy is a new method based on the Huffman coding principle and sample entropy.
(3) We build a multi-class SVM model based on the directed acyclic graph (DAG) principle. We propose an improved adaptive particle swarm optimization (APSO) to train the multi-class SVM model. Compared with other methods, the proposed method has an excellent ability in anomaly detection.

The rest of this paper is organized as follows. Section 2 presents the scheme of the proposed anomaly detection framework. The construction method of multi-type feature sequence HMSE-T/F is provided in Section 3. In Section 4, the anomaly detection method based on multi-class SVM model and the improved adaptive particle swarm optimization (APSO) are stated. In Section 5, the performance of the proposed method is evaluated from different aspects. Finally, in Section 6, a comprehensive summary of this paper and prospects for future work are given.

## 2. The Scheme of the Proposed Anomaly Detection Framework

### 2.1. Description of Difficulties in Spacecraft Anomaly Detection

In fact, since satellites are at normal working conditions at most of the time during their orbits, the proportion of normal data in the telemetry data collected on the ground is very high. For most detection methods that rely on plenty of training data, satellite telemetry data can provide very few abnormal or fault samples, and there are very few effective samples that can be used for classification model training. Therefore, some adaptive improvements are needed when using the classification model to detect anomalies in spacecraft.

**Figure 1:**
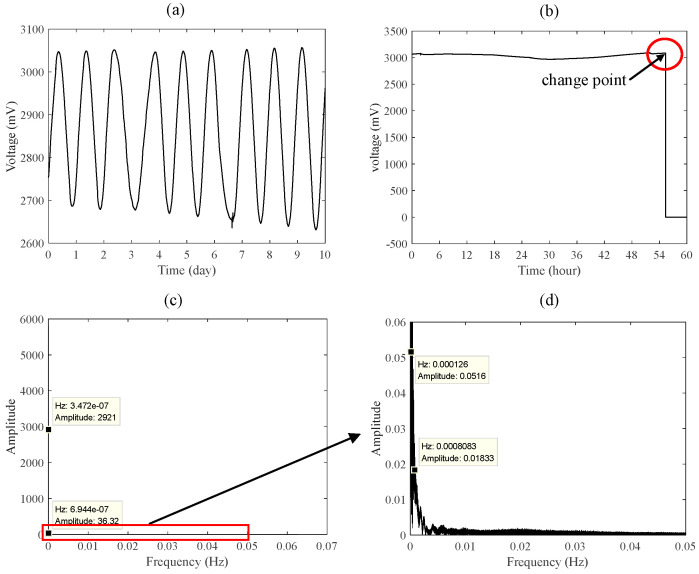
A satellite’s momentum wheel voltage telemetry data: (**a**) 10-day data sampled at a fre-quency of 0.125 Hz, (**b**) sample with sudden change, (**c**) frequency spectrum, (**d**) partially enlarged view of (**c**).

Figure 1a shows the momentum wheel voltage change of a certain type of satellite within 10 days, and its sampling frequency is 0.125 Hz. Figure 1b shows a sudden voltage change in a certain type of satellite. Figure 1c is the frequency spectrum of the telemetry signal in Figure 1a,d is the partially enlarged view of Figure 1c. According to Figure 1a–d, apart from the feature of less abnormal data, satellite telemetry data also exhibits the characteristics of extremely low sampling frequency, slow data change over a long period of time, and many sudden abnormalities. Therefore, anomaly detection methods that rely on time domain and frequency domain feature extraction often find it difficult to distinguish the health status of their telemetry data.

### 2.2. The Proposed Anomaly Detection Framework

**Figure 2:**
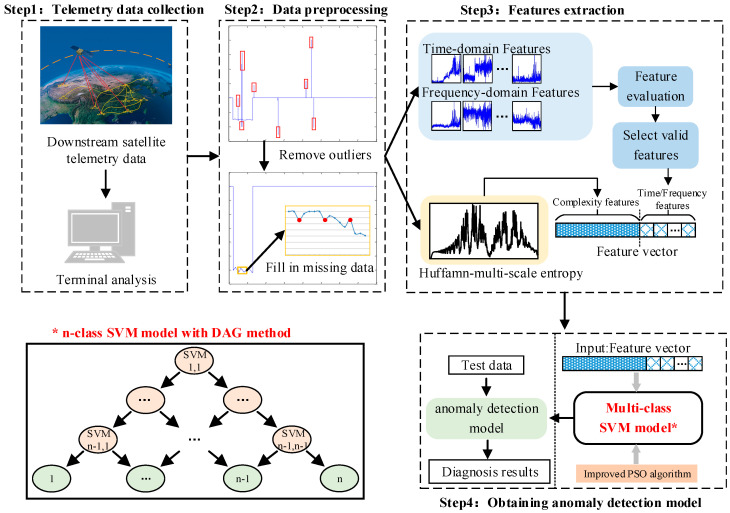
The overall procedure of the proposed anomaly detection framework.

To effectively solve the problem of satellite momentum wheel anomaly detection, a new anomaly detection framework based on multi-type feature extraction and fusion is proposed in this paper. The overall procedure of the proposed anomaly detection framework is shown in Figure 2. Specifically, the descriptions of each Step are detailed as follows.

Step 1: Telemetry data collection.

When the satellite is in orbit, to obtain its internal operating status and further provide real-time data for the remote-control object, the sensors in the satellite telemetry system need to measure the operating status of each key component and convert it into electrical signals. After the signals of each channel are combined according to a certain system, they are transmitted to the ground telemetry equipment (including receiver, antenna and splitter demodulator) using radio communication technology, and the ground terminal equipment restores and stores the original parameter information of each channel through signal demodulation technology.

Step 2: Data preprocessing.

The collection process of telemetry data is interfered with by sensors, converters, and wireless transmission. The data obtained by the ground receiving end often produces abnormal jump points. These kind of data points that deviate from the change law of the measured signal are usually called abnormal outliers. The abnormal outliers of the telemetry data will provide wrong information and affect the processing and analysis results of the telemetry signal. Outlier elimination is an important part of telemetry data preprocessing. By eliminating random measurement values with large errors, the authenticity of telemetry data can be guaranteed to a certain extent, and the reliability of data analysis can be improved. Commonly used methods to eliminate outliers include visual inspection, mean square method, point discrimination, Letts criterion, etc. Different outlier elimination methods should be used for different types of telemetry data. Considering that this article mainly analyzes the telemetry data of the satellite momentum wheel, the outlier elimination method based on the Letts criterion is adopted.

The premise of the Letts criterion is that the distribution of the measured data is close to the normal distribution. Based on this assumption, the given confidence probability is 99.7% as the standard, and the standard deviation of three times the measured quantity is used as the basis. Any measurement value exceeding this limit is judged for wild value. For a given sequence of telemetry measurement values. For a given telemetry sequence $x=\left\{x_{i}\right\},i=1,\cdots ,N$, the specific process of the method is as follows.

(1) Calculate the mean of the series:

(1) $$\bar{x}=\sum_{i=1}^{N}x_{i}$$

(2) Calculate the standard deviation of the series:

(2) $$\sigma =\frac{1}{N}\sqrt{\sum_{i=1}^{N}\left(x_{i}-\bar{x}\right)}$$

(3) Eliminate outliers:

(3) $$\{\begin{array}{lll} \left|x_{i}-\bar{x}\right|\leq 3\sigma , & not\begin{array}{l} outliers, \end{array} & keep\\ \left|x_{i}-\bar{x}\right|>3\sigma , & outliers, & delete \end{array}$$

In addition to the problem of outliers, the process of satellite telemetry data transmission to the ground is affected by the ionosphere, and data may be missing during the signal decoding process. A telemetry sequence that has many data problems should be discarded and not used as training data, but the missing value at a certain point in the sequence can be handled by the filling method. From the distribution of the missing values, they can be divided into missing completely at random (MCAR), missing at random (MAR) and missing not at random (MNAR). MCAR means that the law of missing values in the data is completely random and does not affect the unbiasedness of the overall sample. MAR means that the mechanism of missing data is not completely random. The missing data of this type depends on other variables. Such missing values are relatively rare in telemetry data. MNAR means that the missing data is related to the value of the variable itself.

The missing values in satellite telemetry data are generally MCAR, so this paper uses an interpolation method based on two short sequences before and after the missing point to fill in the missing values. Given the data sequence to be filled is $y=\left\{y_{i}\right\},i=1,\cdots ,M$, The missing value to be filled is $y_{}^{*}$. The auxiliary variable used to construct the regression equation is $x=\left\{x_{i}\right\},j=1,\cdots ,M$. The auxiliary variable value corresponding to the missing value of the variable to be filled is $x^{*}$, and $x^{*}$ is a known variable. Use x,y to construct the regression equation:

(4) $$y_{i}=f^{*}\left(x_{i}\right)$$

where $f^{*}$ needs to choose different regression models according to different telemetry data. Then the missing value is $y^{*}=f\left(x^{*}\right)$.

Step 3: Features extraction.

Considering the difficulty of using satellite telemetry data for anomaly detection as mentioned above, this paper adopts a time-frequency domain feature extraction and selection method based on feature quality evaluation. At the same time, a complexity feature extraction method based on Hoffman multi-scale entropy is proposed, which enriches signal feature types and provides effective feature learning samples for training satellite telemetry data anomaly detection models. The specific method of feature extraction is described in detail in Section 3.

Step 4: Obtaining the anomaly detection model.

This paper takes support vector machine (SVM) as the basic unit and uses a directed acyclic graph (DAG) principle to construct a satellite momentum wheel anomaly detection model based on the support vector machine. This model can effectively solve the multi-classification problem when some categories are difficult to distinguish. In addition, to improve the classification accuracy of the anomaly detection model, an improved particle swarm optimization (PSO) algorithm is proposed to train SVMs. The specific method of Obtaining anomaly detection model is described in detail in Section 4.

## 3. Multi-Type Feature Sequence HMSE-T/F Construction Method

### 3.1. Time/Frequency Domain Feature Extraction and Selection

#### 3.1.1. Time/Frequency-Domain Feature

The time domain signal is a time series in which time is the independent variable to describe the change of a certain physical quantity, and it is the most basic and most intuitive form of expression of the signal. The time domain signal reflects the corresponding relationship between real physical information and time. The processing of filtering, amplifying, statistical feature calculation, and correlation analysis of signals in the time domain is collectively referred to as time domain analysis.

When a device fails, its spectrum distribution may change. Like the statistical analysis of time-domain signals, this type of change can be described by statistical analysis of the signal’s frequency spectrum.

Given a period of time domain signal x(t), the frequency spectrum of this signal is y(k),k=1,⋯,k, $f_{k}$ is the k-th line of the spectrum. Then the time-domain statistical characteristics and frequency-domain statistical features of x(t) are shown in Table 1 [28].

#### 3.1.2. Feature Evaluation and Selection

In this paper, two commonly used feature evaluation methods, Laplacian Score (LS) [29] and Relief-F Score (RFS) [30], are used to evaluate the effectiveness of the time-domain and frequency-domain features of the satellite momentum wheel telemetry signal. Feature selection is based on two different feature evaluation results, and the feature with the higher evaluation score is taken as the effective feature in the time/frequency domain.

(1) Laplacian Score (LS).

In practical problems, data of the same type are generally close to each other. Under this premise, the importance of describing features can be transformed into evaluating the local retention of features. The Laplace score is based on this idea. Let the data set be $X\in {\mathbb{R}}^{m\times n}$, $L_{r}$ is the LS of the r-th feature, $f_{ri}$ is the the r-th feature of the i-th sample. $L_{r}$ can be calculated as follows.

Step 1: Construct a neighbor graph G containing n nodes, the i-th node corresponds to the i-th sample $x_{i}$, if $x_{i}$ and $x_{j}$ are close to each other, that is, $x_{i}$ is within the k-neighbor range of $x_{j}$, then an edge is constructed between nodes $x_{i}$ and $x_{j}$. When the data labels are known, edges can be constructed directly between samples of the same type.

Step 2: If nodes $x_{i}$ and $x_{j}$ are connected, put $S_{ij}=e^{\frac{{\left\|x_{i}-x_{j}\right\|}^{2}}{t}}$, where t is a suitable constant. Otherwise, put $S_{ij}=0$. The weight matrix S of the graph models the local structure of the data space.

Step 3: For the r-th feature, the $f_{r}$ and D can be defined as $f_{r}={\left[f_{r1},f_{r2},\cdots ,f_{rm}\right]}^{T}$, D=diag(S1). The matrix L=D−S is often called graph Laplacian. Let

(5) $${\tilde{f}}_{r}=f_{r}-\frac{f_{r}^{T}D1}{1^{T}D1}1$$

where $1={\left[1,\cdots ,1\right]}^{T}$.

Step 4: Compute the LS of the r-th feature as follows:

(6) $$L_{r}=\frac{{\tilde{f}}_{r}^{T}L{\tilde{f}}_{r}}{{\tilde{f}}_{r}^{T}D{\tilde{f}}_{r}}$$

(2) Relief-F Score (RFS).

The Relief-F Score method is a multi-class variant of the Relief method. The Relief method designs a correlation statistic to measure the importance of features. The statistic is a vector, each component of which corresponds to an initial feature, and the importance of the feature subset is determined by the sum of the relevant statistic components corresponding to each feature in the subset. For each $x_{i}$ in the data set $X\in {\mathbb{R}}^{m\times n}$, first find its nearest neighbor $x_{i,nh}$ in the same sample of $x_{i}$, which is called guessing nearest neighbor, and then find its nearest neighbor $x_{i,nm}$ from different type samples of $x_{i}$, which is called guessing wrong neighbor. The component of the correlation statistic corresponding to the feature is:

(7) $$\delta^{\left(r\right)}=\sum_{i}\left(-diff{\left(x_{i}^{\left(r\right)},x_{i,nh}^{\left(r\right)}\right)}^{2}+diff{\left(x_{i}^{\left(r\right)},x_{i,nm}^{\left(r\right)}\right)}^{2}\right)$$

where $x_{i}^{\left(r\right)}$ is the value of the r-th feature of $x_{i}$. For $x_{a}$ and $x_{b}$, $diff\left(x_{a}^{\left(r\right)},x_{b}^{\left(r\right)}\right)$ depends on the type of the r-th feature. If the r-th feature r is discrete, when $x_{a}^{\left(r\right)}=x_{b}^{\left(r\right)}$, $diff\left(x_{a}^{\left(r\right)},x_{b}^{\left(r\right)}\right)=0$, otherwise $diff\left(x_{a}^{\left(r\right)},x_{b}^{\left(r\right)}\right)=1$. If the r-th feature r is continuous, then $diff\left(x_{a}^{\left(r\right)},x_{b}^{\left(r\right)}\right)=\left|x_{a}^{\left(r\right)}-x_{b}^{\left(r\right)}\right|$.

Relief is designed for two classification problems, while Relief-F can handle multiple classification problems. For the sample $x_{i}$, if it belongs to the k-th class, the Relief-F method first finds its nearest neighbor $x_{i,nh}$ in the k-th class sample, and then finds a nearest neighbor $x_{i,l,nm}^{},l\neq k$ of $x_{i}$ in each class except the k-th class as a guessing wrong neighbor, so the correlation statistic corresponding to the component of the r-th feature is

(8) $$\delta^{\left(r\right)}=\sum_{i}-diff{\left(x_{i}^{\left(r\right)},x_{i,nh}^{\left(r\right)}\right)}^{2}+\sum_{l\neq k}\left(p_{l}\times diff{\left(x_{i}^{\left(r\right)},x_{i,nm}^{\left(r\right)}\right)}^{2}\right)$$

where $p_{l}$ is the proportion of the l-th class sample in the data set X.

### 3.2. Complexity Features Based on Huffman-Multi-Scale Entropy (HMSE)

Sample entropy (SampEn) is a new time series complexity characterization parameter proposed by Richman et al. in 2004 [31]. The sample entropy is improved on the basis of approximate entropy, both of which measure the complexity of the time series and the probability of a new pattern generated by the sequence when the dimensionality changes. The greater the probability of generating a new pattern, the more complex the sequence and the higher the entropy value. Compared with other nonlinear dynamic methods such as Lyapunov exponent, information entropy, and correlation dimension, sample entropy has the advantages of short data, strong anti-noise and anti-interference ability, and good consistency within a large range of parameters. Therefore, it has attracted the attention of many scholars and has been frequently used in the field of mechanical signal analysis and fault diagnosis in recent years.

#### 3.2.1. Traditional Multi-Scale Sample Entropy (MSE)

Suppose a time series of length *N* is $X=\{x_{1},x_{2},\cdots ,x_{N-1},x_{N}\}$, and the calculation method of sample entropy is as follows:

Step 1: Construct the time series X into an *m*-dimensional vector:

(9) $$X(i)=\{x_{i},x_{i+1}\cdots ,x_{i+m-1}\},\;i=1,2,\cdots ,N-m+1$$

Step 2: Define the distance between X(i) and X(j) as d[X(i),X(j)], (i≠j), which is the largest difference between the two corresponding elements:

(10) $$d[X(i),X(j)]=\underset{k\in (0,m-1)}{\max}|x(i+k)-x(j+k)|,\;(i\neq j)$$

Step 3: Given a threshold r>0, count the number of d[X(i),X(j)]<r and calculate the ratio to the total number of vectors N−m:

(11) $$B_{i}^{m}(r)=\frac{1}{N-m}num\{d[X(i),X(j)]<r\}$$

Step 4: Average all the results obtained by Equation (12):

(12) $$B^{m}(r)=\frac{1}{N-m+1}\sum_{i=1}^{N-m+1}B_{i}^{m}(r)$$

Step 5: Then m = m + 1, repeat Step1–Step4.

Step 6: Then theoretically the sample entropy of this sequence is:

(13) $$SampEn(m,r)=\underset{N\to \infty }{\lim}\{-\ln(\frac{B^{m+1}(r)}{B^{m}(r)})\}$$

However, *N* cannot be infinite in fact, but a finite value. The estimated value of sample entropy is:

(14) $$SampEn(m,r,N)=-\ln(\frac{B^{m+1}(r)}{B^{m}(r)})$$

The sample entropy does not include the comparison of its own data segments, which not only improves the calculation accuracy and saves the calculation time, but also makes the calculation of the sample entropy independent of the data length. In addition, the sample entropy has better consistency. In other words, if one sequence has a higher SampEn than another sequence, then when the parameters *m* and *r* are changed, the sequence still has a relatively high SampEn value. However, the disadvantage of sample entropy is that it does not consider the different time scales that may exist in the time series.

To calculate the complexity of the signal at different time scales, Costa et al. proposed multi-scale entropy [32], which aims to extend the sample entropy to multiple time scales to provide additional observation perspectives when the time scale is uncertain. Like other entropy measurement methods, the goal of multi-scale entropy is to evaluate the complexity of time series. One of the main reasons for using multi-scale entropy is that the relevant time scale in the time series is not known. For example, when analyzing a speech signal, it is more effective to count the complexity of the signal under the word time scale than the complexity of the entire speech segment. However, the actual situation is that we often cannot know how many words a certain speech segment contains, or know what time scale should be used to obtain more useful information from the original signal. Therefore, analyzing the problem through multiple time scales will obtain more effective information.

**Figure 3:**
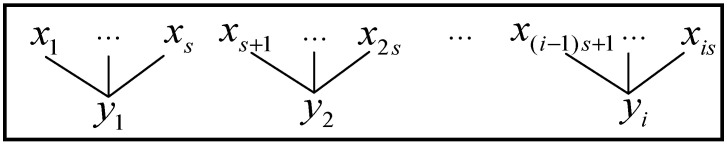
The process of coarse-graining the time series.

The basic principle of multi-scale entropy (MSE) includes coarse-graining or down-sampling the time series, so that the time series can be analyzed at increasingly coarse time resolutions. Given a time series $X=\{x_{1},x_{2},\cdots ,x_{N-1},x_{N}\}$ of length *N*, set the coarse-grained scale to s, then the original time series can be split into *i* consecutive segments without overlap, where i=floor(N/s), floor(*) means taking the largest integer smaller than *. The original sequence can be transformed into a new sequence by calculating the average value of each fragment by Equation (15). Then the MSE of the original sequence can be obtained by solving the sample entropy of the new sequence $Y=\{y_{1},y_{2},\cdots ,y_{i}\}$ obtained under different s. The process of coarse-graining the time series is shown in Figure 3.

(15) $$y_{i}=\frac{\sum_{k=1}^{s}x_{(i-1)s+k}}{s}$$

#### 3.2.2. The Huffman-Multi-Scale Entropy (HMSE)

**Figure 4:**
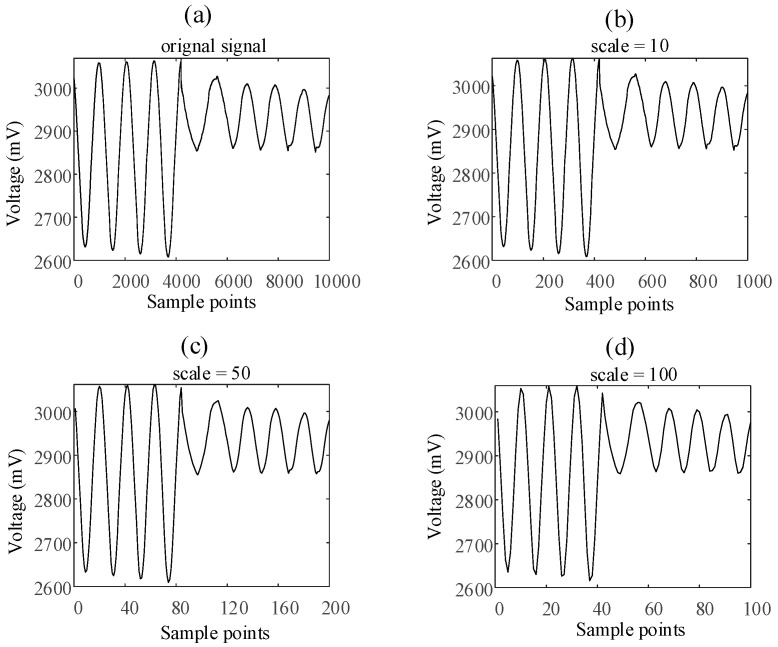
The average value of voltage telemetry under different scales: (**a**) original signal, (**b**) scale = 10, (**c**) scale = 50, (**d**) scale = 100.

According to the process of the calculation of multi-scale sample entropy, the core of this method is to coarse-grain the original time series on different time scales by averaging. Figure 4a shows a satellite momentum wheel voltage telemetry signal with the length of 10,000. This signal is coarse-granulated and averaged on time scales s=10, s=50, and s=100 respectively. The results are shown in Figure 4b–d. As can be seen from Figure 4, the waveform of the new signal obtained by averaging the original signal at different time scales is almost the same.

It can be seen from Figure 4 that the state of the signal changes at about the 4000th sample point in the original signal. However, the use of different coarse-grained scales cannot reflect the difference in signal changes. Therefore, this paper proposes a new improved multi-scale entropy calculation method based on the Huffman mean model. The main innovation of this method is that when the original data is coarse-grained on different time scales, the average value is not taken, but the Huffman average value is taken. This section will introduce the Huffman mean model and the improved multi-scale entropy calculation method based on the Huffman mean model in detail.

(1) Huffman Coding.

In 1952, Huffman proposed an optimum method of coding an ensemble of messages consisting of a finite number of members [33]. A minimum-redundancy code is one constructed in such a way that the average number of coding digits per message is minimized. The process of Huffman coding is as follows.

Step 1: Given a sequence containing *n* kinds of symbols. Suppose the set of symbol types is $S_{0}=\{s_{1}^{0},s_{2}^{0},\cdots ,s_{i}^{0},\cdots s_{n}^{0}\},i=1,2,\cdots ,n$. The probability of each symbol appearing is $P_{0}=\{p_{1}^{0},p_{2}^{0},\cdots ,p_{i}^{0},\cdots p_{n}^{0}\},i=1,2,\cdots ,n$, and $\sum_{i=1}^{n}p_{i}^{0}=1$.

Step 2: Set the iteration parameter to t, the maximum value of t is n−1 and the initial value of t is 0. The symbol sequence and the corresponding probability at the beginning of the *k*-th iteration are $S_{k-1}$ and $P_{k-1}$. The symbol sequence and the corresponding probability at the end of the *k*-th iteration are $S_{k}$ and $P_{k}$.

Step 3: When t=k, arrange the symbol $S_{k-1}$ in ascending order of probability $P_{k-1}$ as $S_{k-1}=\{s_{1}^{k-1},s_{2}^{k-1},\cdots ,s_{i}^{k-1},\cdots s_{n-k+1}^{k-1}\}$. Then the probability $P_{k-1}$ is also rearranged accordingly as $P_{k-1}=\{p_{1}^{k-1},p_{2}^{k-1},\cdots ,p_{i}^{k-1},\cdots p_{n-k+1}^{k-1}\}$.

Step 4: Take the two symbols $s_{1}^{k-1}$ and $s_{2}^{k-1}$ with the least probability in the symbols sequence. Encode the symbol $s_{2}^{k-1}$ with higher probability into “1” and the symbol $s_{1}^{k-1}$ with lower probability as “0”. Add the probabilities $p_{1}^{k-1}$ and $p_{2}^{k-1}$ of the $s_{1}^{k-1}$ and $s_{2}^{k-1}$ as the probability $p^{*}$ of the new symbol $s^{*}$.

Step 5: Delete $s_{1}^{k-1}$ and $s_{2}^{k-1}$ from $S_{k-1}$, and add $s^{*}$ into $S_{k-1}$. Then the $S_{k-1}$ turns into $S_{k}$, and the size of $S_{k}$ is n−k. Delete $p_{1}^{k-1}$ and $p_{2}^{k-1}$ from $P_{k-1}$, and add $p^{*}$ into $P_{k-1}$. Then the $P_{k-1}$ turns into $P_{k}$, and the size of $P_{k}$ is also n−k.

Step 6: Repeat the Step 3 to Step 5 until t=n−1. Then the symbols sequence will be $S_{n-1}=\{s_{1}^{n-1}\}$ and the probability will be $P_{n-1}=\{p_{1}^{n-1}\},p_{1}^{n-1}=1$.

It can be seen from the above coding process that the symbol with the lower probability in the original signal has the longer Huffman code length. Conversely, the symbol with the higher probability has the shorter Huffman code length. The complexity of the probability distribution of the signal can be described by solving the Huffman average code length of the original signal. Based on the above-mentioned Huffman coding process, the method to further calculate the average Huffman coding length is as follows.

Backtrack from the symbol $s_{1}^{n-1}$ with the probability of $p_{1}^{n-1}=1$ to each source symbol and record 0/1 in the backtracking path. The Huffman code of $s_{i}^{0}$ is $c_{i}$. The average Huffman coding length $L^{*}$ can be calculated according to the length of $c_{i}$ and the corresponding probability $p_{i}^{0}$ as Equation (16). $L(c_{i})$ is the length of $c_{i}$.

(16) $$L^{*}=\sum_{i=1}^{n}p_{i}^{0}*L(c_{i})$$

For a set of source symbols $S_{0}=\{s_{1},s_{2},s_{3},s_{4},s_{5},s_{6}\}$ with probability $P_{0}=\{0.35,0.28,0.14,0.13,0.07,0.03\}$, the process of Huffman coding is shown in Table 2. The average Huffman coding length of $S_{0}$ can be calculated as 2.33 as follows.

$$L^{*}(S_{0})=(0.35+0.28+0.14)*2+0.13*3+(0.07+0.03)*4=2.33$$

(2) Huffman Mean Model.

The basic principle of Huffman coding and the calculation method for solving the average Huffman coding length were introduced above. In this paper, a new Huffman mean model based on the Huffman coding is proposed for the problem of satellite anomaly detection. For a sequence $T=\{t_{1},t_{2},\cdots ,t_{i},\cdots ,t_{n}\}$, the expression of the Huffman mean model is shown in Equation (17).

(17) $$HM(T)=\{\begin{array}{l} T^{\prime }=T/sum(T)\\ C=Huffman\_coding(T^{\prime })\\ \ell =L(C)\\ Huffman\_mean=sum(T*(\ell /sum(\ell ))) \end{array}$$

where HM(T) is the Huffman mean value of T, $T^{\prime }=T/sum(T)$ means to convert the original time series into a probability series, $C=Huffman\_coding(T^{\prime })$ represents the Huffman coding result of the probability sequence $T^{\prime }$, $C=\{c_{1},c_{2},\cdots ,c_{i},\cdots ,c_{n}\},i=1,2,\cdots ,n$, $c_{i}$ is the Huffman code corresponding to $t_{i}$ in the original sequence, ℓ=L(C) means to calculate the length of each $c_{i}$, Huffman_mean=sum(T*(ℓ/sum(ℓ))) represents the Huffman mean value of the sequence T considering the length weight of the Huffman code.

(3) The Improved Method of Huffman-multi-scale Entropy.

**Figure 5:**
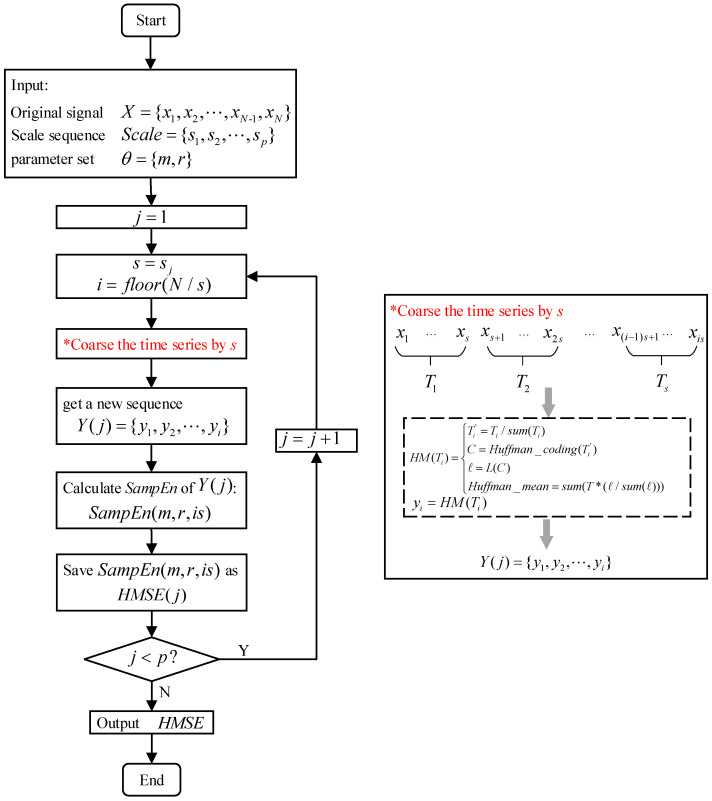
The process of the improved method of Huffman-multi-scale Entropy.

The Figure 5 shows the calculation process of Huffman-multi-scale entropy. The inputs of both two methods are original signal $X=\{x_{1},x_{2},\cdots ,x_{N-1},x_{N}\}$, the scale sequence $Scale=\{s_{1},s_{2},\cdots ,s_{p}\}$ and the parameter set θ={m,r}, usually 0.1std(X)<r<0.2std(X). Compared with the classic MSE, the Huffman-multi-scale entropy method proposed in this paper adopts the coarse-grained method based on the Huffman mean model.

**Figure 6:**
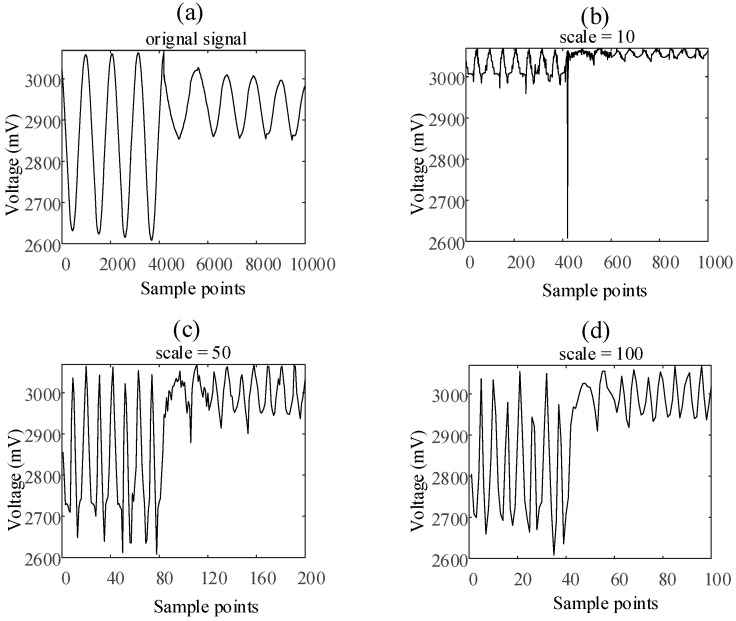
The Huffman average code length of voltage telemetry under different scales: (**a**) original signal, (**b**) scale = 10, (**c**) scale = 50, (**d**) scale = 100.

Figure 6 shows the same satellite momentum wheel voltage telemetry signal with the length of 10,000. This signal is coarse-granulated and calculated by Huffman mean model on time scales s=10, s=50, and s=100, respectively. Obviously, the coarse-grained method based on the Huffman mean model can enhance the difference of signal changes at different time scales.

## 4. Anomaly Detection Method Based on Multi-Class SVM

### 4.1. Multi-Class SVM

A support vector machine (SVM) is widely used in classification problems. The basic idea is to find a hyperplane so that all sample points in the positive and negative categories are farthest from the plane, and points that are far enough from the plane can basically be correctly classified. Therefore, if the points closer to the hyperplane are as far away as possible from the hyperplane, a better classification effect can be achieved.

This article uses the most interval classifier to achieve two-class SVM, and then uses the directed acyclic graph (DAG) method to achieve the multi-class SVM based on two-class SVM.

Set the dataset as $\left\{(x_{i},y_{i})|i=1,2,\cdots ,N\},x_{i}\in R_{n},y\in \{-1,+1\right\}$, the hyperplane is $w^{T}x+b=0$, Then the distance from the support vector to the hyperplane is $w^{T}x+b=y$, which can be written as

(18) $$\frac{\left|y(w^{T}x+b)\right|}{||w|{|}_{2}}=\frac{1}{||w|{|}_{2}}$$

The SVM model keeps all the points on both sides of the support vector of their respective categories, while keeping away from this hyperplane. It can be seen from Equation (18) that when $||w|{|}_{2}$ is the smallest, the interval is the largest. Introduce the penalty parameter λ for misclassification and the relaxation factor ξ that allows misclassification, and the objective function can be

(19) $$\begin{array}{c} \min\frac{1}{2}||w|{|}_{2}^{2}+\lambda \sum_{i=1}^{N}\xi_{i}\\ s.t.y^{i}(w^{T}x+b)\geq 1-\xi_{i},i=1,2,\cdots ,N\\ \xi_{i}\geq 0,i=1,2,\cdots ,N \end{array}$$

According to Lagrange’s duality, the optimization objective can be converted into an equivalent dual problem. The Equation (19) can be transformed into:

(20) $$\begin{array}{c} \underset{\alpha }{\min}\frac{1}{2}\sum_{i,j=1}^{N}y_{i}y_{j}\alpha_{i}\alpha_{j}K\langle x_{i},x\rangle -\sum_{j=1}^{N}\alpha_{j}\\ s.t.y^{i}(w^{T}x+b)\geq 1-\xi_{i},i=1,2,\cdots ,N\\ \xi_{i}\geq 0,i=1,2,\cdots ,N \end{array}$$

where $K\langle x_{i},x\rangle$ is the kernel function. The radial basis function is used as the kernel function in this paper, $K\langle x_{i},x\rangle =\exp\{-\frac{||x_{i}-x|{|}^{2}}{2\sigma^{2}}\}$, σ is the kernel function parameter. Then the decision function is

(21) $$f(x)=w^{T}x+b=\mathrm{sgn}[\sum_{i=1}^{N}y_{i}\alpha_{i}K\langle x_{i},x\rangle +b],0<\alpha_{i}<\lambda$$

Among the multi-class SVM methods, one is the direct solution method, but this method has high time complexity and is difficult to implement. It is not suitable for a large amount of data. The other one is to combine multiple two-class SVM models into a multi-class SVM model. In this paper, a directed acyclic graph method is used to construct a multi-class SVM.

**Figure 7:**
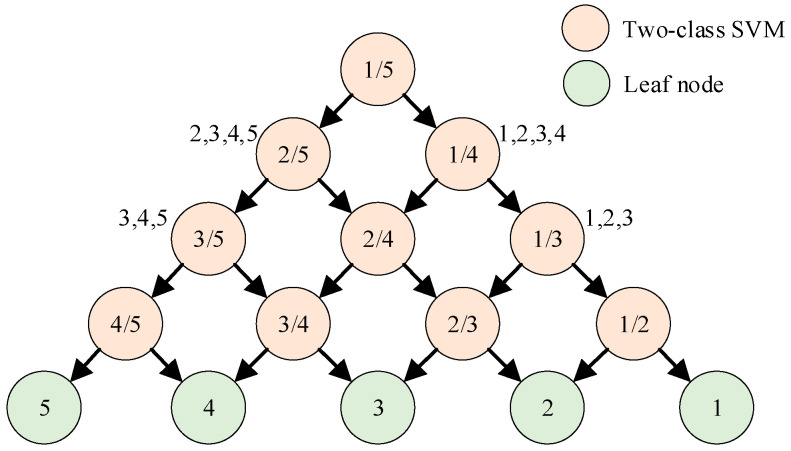
The schematic diagram of DAG method for five-class SVM model.

The DAG method uses the “competition” rule. For n types, the height of the decision tree is n−1. Put the classes that are easy to distinguish on the upper layer, and the classes that are difficult to distinguish on the lower layer. The schematic diagram of DAG method for five-class SVM is shown in Figure 7.

### 4.2. Improved Adaptive Particle Swarm Optimization (APSO)

Kennedy and Eberhart first proposed particle swarm optimization (PSO) in 1995 [34]. PSO is an algorithm for finding the optimal solution inspired by the foraging behavior of bird groups. In the PSO algorithm, each particle represents a feasible solution of a function to be optimized, and the movement of the particle is restricted by two aspects: speed and position. The speed constrains the distance of particle movement, while the position constrains the direction of particle movement. Each particle’s movement is given a fitness function to evaluate the particle’s location. Under the control of constraint conditions and evaluation function, the particles search for a better area in the process of moving. After many iterations, they gather near the optimal solution. The particle velocity and position update formula are as follows:

(22) $$v_{id}^{k+1}=\omega v_{id}^{k}+c_{1}r_{1}(p_{id}-x_{id}^{k})+c_{2}r_{2}(p_{gd}-x_{id}^{k})$$

(23) $$x_{id}^{k+1}=x_{id}^{k}+v_{id}^{k+1}$$

where $v_{id}^{k}$ is the current velocity of the *d*-th component in the *i*-th particle, $v_{id}^{k+1}$ is the next velocity of the *d*-th component in the *i*-th particle, ω is the inertia weight, ω≥0, $c_{1}$ and $c_{2}$ are the acceleration constant of the particle, $r_{1}$ and $r_{2}$ are random numbers between 0 and 1, $r_{1},r_{2}=random(0,1)$, $p_{id}$ represents the best position of the *d*-th component of the *i*-th particle, $p_{gd}$ represents the best position of the *d*-th component of all particles, $x_{id}^{k}$ is the current position of the *d*-th component in the *i*-th particle, and $x_{id}^{k+1}$ is the next position of the *d*-th component in the *i*-th particle.

PSO has the advantages of fewer parameters and fast convergence, but it also has shortcomings such as premature convergence and falling into local optimum. It can be seen from Equations (22) and (23) that the inertia weight ω determines the relationship between the next flight distance and the current flight distance, which further affects the position after the flight. The larger the ω, the stronger the particle’s flying ability in the solution space, which is conducive to searching in the global scope. The smaller the ω, the smaller the flight length, and the stronger the search ability of the particles in a local area, which is conducive to the convergence of the algorithm. However, if the value of ω is too large, it will easily cause the algorithm to skip the optimal solution or oscillate near the optimal solution, which will lead to the premature convergence; if ω is too small, the algorithm will easily fall into a local optimum.

The inertia weight ω should be a larger value at the beginning of the iteration to ensure a strong global search ability and the ability to jump out of the local optimum. However, in the later stage of the algorithm iteration, smaller *ω* should be used to ensure strong local search capabilities, which is conducive to the convergence of the algorithm.

In response to the above problem, this paper proposes a strategy for adaptively changing the ω according to the number of iterations and the current fitness value. The formula is as follows:

(24) $$\omega_{id}^{k+1}=\{\begin{array}{l} \omega_{0}-e^{-k/K}*\frac{f_{\max}^{k}-f_{id}^{k}}{f_{\max}^{k}-f_{avg}^{k}},f_{id}^{k}\leq f_{avg}^{k}\\ \omega_{0}+e^{-k/K}*\frac{f_{id}^{k}-f_{\min}^{k}}{f_{avg}^{k}-f_{\min}^{k}},f_{id}^{k}>f_{avg}^{k} \end{array}$$

where k is the current number of iterations, k+1 is the next number of iterations, K is the maximum number of iterations, $\omega_{id}^{k+1}$ is the inertia weight for the next iteration for the *d*-th component of the *i*-th particle, $\omega_{0}$ is the initial value of ω, $\omega_{0}=0.5$ in this paper, $f_{id}^{k}$ is the fitness value of the *d*-th component of the *i*-th particle obtained in the *k*-th iteration, $f_{\max}^{k}$ is the maximum fitness value in the *k*-th iteration, $f_{\min}^{k}$ is the minimum fitness value in the *k*-th iteration, $f_{avg}^{k}$ is the average fitness value in the *k*-th iteration.

At the beginning of the iteration, the weight of the particle changes greatly, and as the number of iterations of the particle increases, the weight change decreases. At the same time, the weight change is determined by the fitness function. When the particle fitness is less than or equal to the average fitness, that is, when the accuracy of the classification model is greater than or equal to the average accuracy, the inertia weight decreases; when the particle fitness is greater than the average fitness, the accuracy of the classification model is lower than the average accuracy, and the inertia weight increases.

The increase or decrease of the inertia weight is determined by the number of iterations. At the beginning of the iteration, the increase or decrease of the weight is large, which is convenient for searching in the global and optimal solution neighborhood. At the later stage of the iteration, the increase or decrease of the weight is small. The increase of the weight can avoid falling into the local optimal solution for random search, and the decrease of the weight facilitates the local fine search.

### 4.3. The Algorithm of the Proposed APSO-SVM

In this paper, the improved Adaptive Particle Swarm Optimization (APSO) is used to optimize the penalty factor λ and the kernel function parameter σ in the SVM. The specific steps of APSO-SVM are as follows.

Step 1: Input the dataset with labels.

Step 2: Divide the dataset into training set and test set, then normalize both two sets.

Step 3: Population initialization. Set the number of particles in the initial population as n. Set the range of penalty factor λ to $\left[\lambda_{\min},\lambda_{\max}\right]$. Set the range of kernel function parameter σ to $\left[\sigma_{\min},\sigma_{\max}\right]$. Initialize the parameter set $\theta =\{\omega_{0},c_{1},c_{2},K\}$. Initialize the position $x_{i}^{0}$, the speed $v_{i}^{0}$, the optimal position $p_{id}$ of *i*-th particle and the global optimal position $p_{gd}$. Set fitness error ε.

Step 4: Calculate the corresponding inertia weight $\omega_{id}^{k+1}$ according to Equation (24) in the adaptive adjustment strategy. Update the velocity $v_{id}^{k+1}$ and position $x_{id}^{k+1}$ of the particles according to Equations (22) and (23). Determine whether λ and σ are in $\left[\lambda_{\min},\lambda_{\max}\right]$ and $\left[\sigma_{\min},\sigma_{\max}\right]$ respectively. If $\lambda <\lambda_{\min}$, set $\lambda =\lambda_{\min}$. If $\lambda >\lambda_{\max}$, set $\lambda =\lambda_{\max}$. If $\sigma <\sigma_{\min}$, set $\sigma =\sigma_{\min}$. If $\sigma >\sigma_{\max}$, set $\sigma =\sigma_{\max}$.

Step 5: If $f_{i}>f(p_{i})$ or $\left|f_{i}-f(p_{i})|\leq \varepsilon ,\lambda (x_{i})<\lambda (p_{i}\right)$, update $p_{i}$. If $f_{i}>f(p_{g})$ or $\left|f_{i}-f(p_{g})|\leq \varepsilon ,\lambda (x_{i})<\lambda (p_{g}\right)$, update $p_{g}$. The expression of f(*) is shown in Equation (21).

Step 6: If k<K, repeat the step 4 to step 6. If k≥K, end the APSO.

Step 7: Use the optimal solution $\left(\lambda^{*},\sigma^{*}\right)$ to create the SVMs model and use this model for classification.

**Figure 8:**
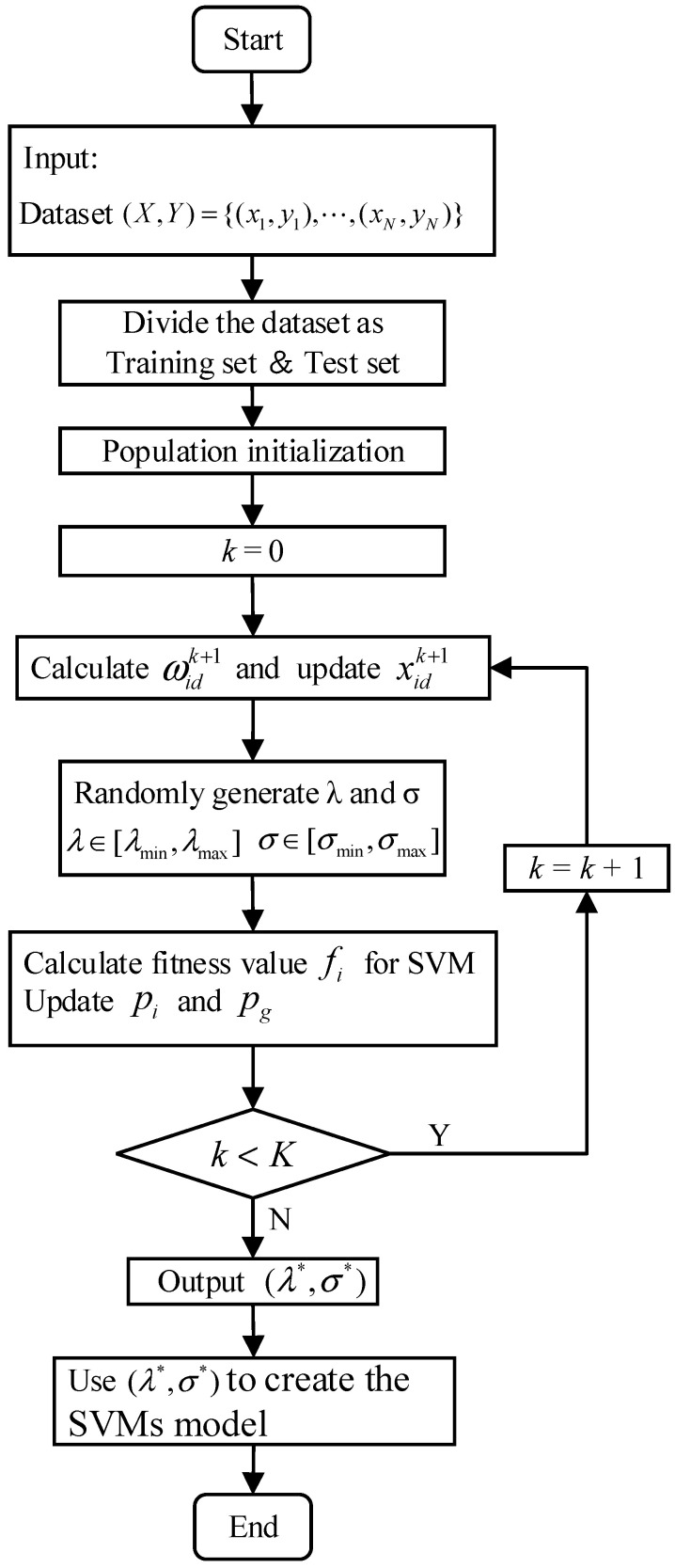
Flow chart of APSO-SVM.

The flow chart of APSO-SVM is shown in Figure 8.

## 5. Case Study of Anomaly Detection

### 5.1. Data Description

**Figure 9:**
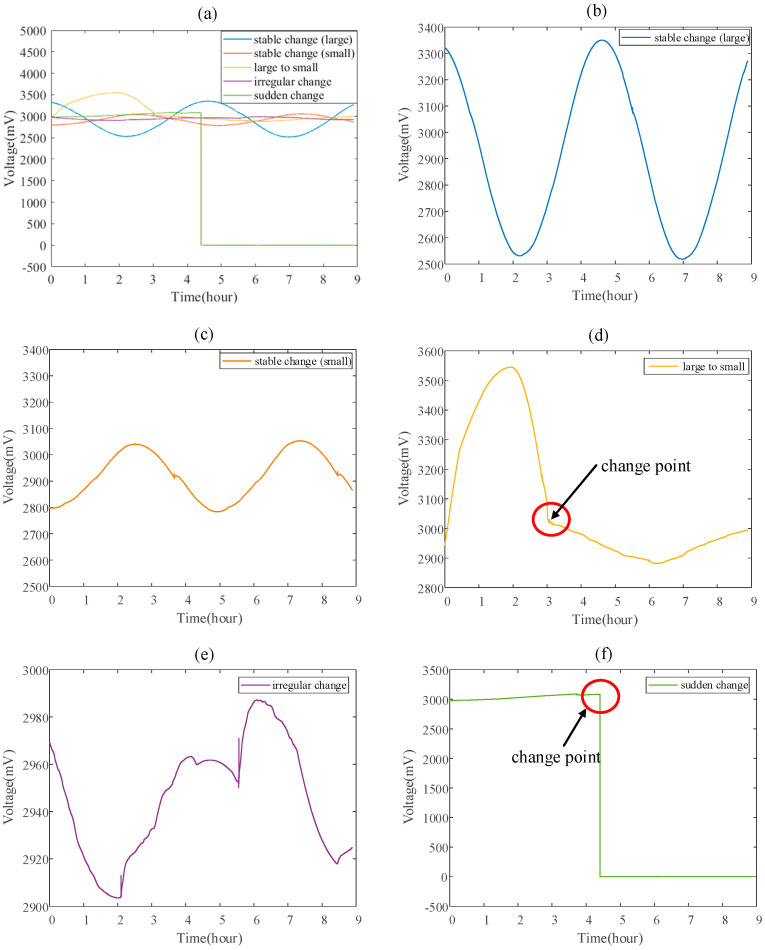
The waveform of five different types of voltage telemetry data for the momentum wheel: (**a**) comparison of 5 types of data in time-domain, (**b**) the waveform of Stable Change (large), (**c**) the waveform of Stable Change (small), (**d**) the waveform of Large to Small, (**e**) the waveform of Irreg-ular Change, (**f**) the waveform of Sudden Change.

The data set used in this article is from a satellite’s telemetry voltage value of its momentum wheel. In this data set, five types of sample with different health status are screened out. Stable Change (large) indicates that the momentum wheel voltage value continuously and steadily changes with a large change amplitude. Stable Change (small) indicates that the momentum wheel voltage value continuously and steadily changes with a small change amplitude. Large to Small indicates that the amplitude of the momentum wheel voltage change smoothly transitions from large to small. The above three types of sample all represent that the momentum wheel is in a normal state. Irregular Change indicates that the voltage of the momentum wheel changes irregularly. Sudden Change indicates that the voltage of the momentum wheel has a sudden change, such as the voltage suddenly jumping to 0. Irregular Change and Sudden Change represent that the momentum wheel is in an abnormal state. The time-domain waveforms of different types of data are shown in the Figure 9.

To verify the effectiveness of the method proposed in this article, the training set and test set used in this article are shown in the Table 3.

### 5.2. Feature Extraction and Selection

#### 5.2.1. Time/Frequency Domain Feature Extraction and Selection

**Figure 10:**
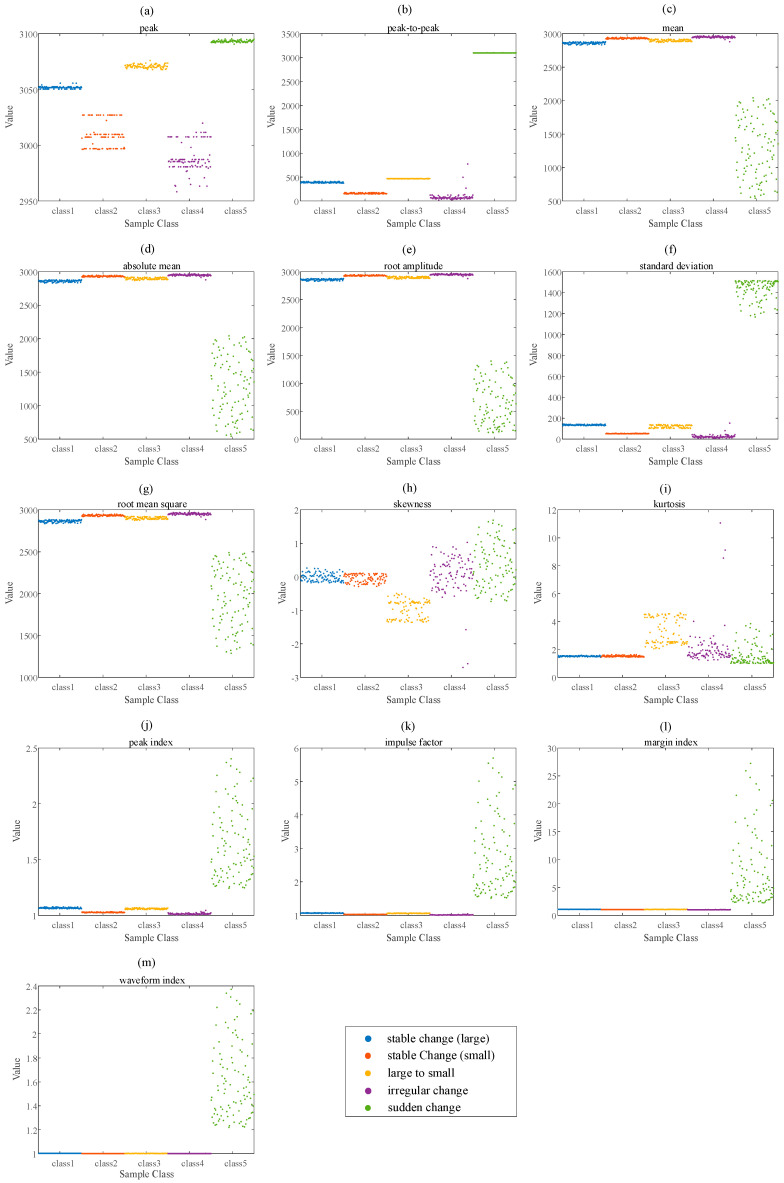
The time-domain features of the five types of momentum wheel voltage telemetry data: (**a**) peak, (**b**) peak-to-peak, (**c**) mean, (**d**) absolute mean, (**e**) root amplitude, (**f**) standard deviation, (**g**) root mean square, (**h**) skewness, (**i**) kur-tosis, (**j**) peak index, (**k**) impulse factor, (**l**) margin index, (**m**) waveform index.

**Figure 11:**
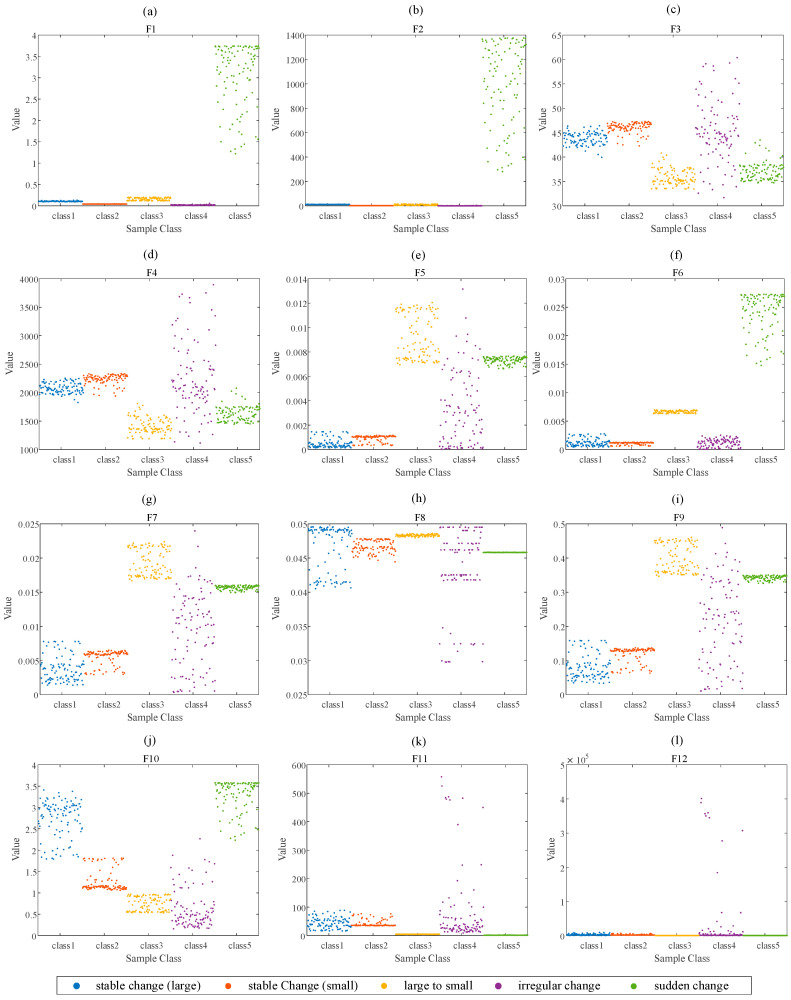
The frequency-domain features of the five types of momentum wheel voltage telemetry data: (**a**) F1, (**b**) F2, (**c**) F3, (**d**) F4, (**e**) F5, (**f**) F6, (**g**) F7, (**h**) F8, (**i**) F9, (**j**) F10, (**k**) F11, (**l**) F12.

According to the time-domain feature and frequency-domain feature calculation methods shown in Table 1, the time-frequency feature values of the five types of momentum wheel voltage telemetry data are calculated. The time-domain features are shown in Figure 10, and the frequency-domain features are shown in Figure 11.

According to the time-frequency feature statistical feature distribution diagrams of different types of data in Figure 10 and Figure 11, the time-domain feature distribution of SC is very scattered, but the frequency-domain feature distribution is relatively more concentrated. Intuitively, peak and peak-to-peak in the time domain feature can distinguish five types of data to a certain extent, and F3 and F4 in the frequency domain feature can also distinguish five types of sample to a certain extent.

**Figure 12:**
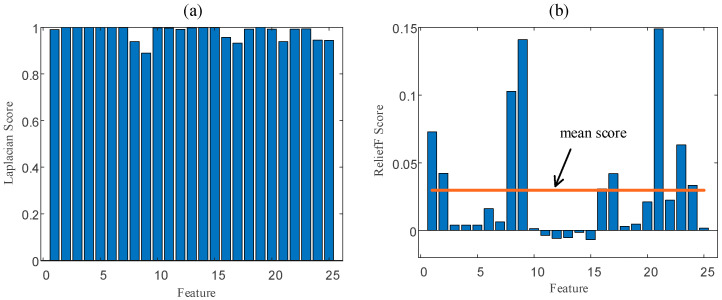
The time-domain (**a**) and frequency-domain (**b**) features evaluation results.

In order to quantify the ability of different feature values to distinguish samples, we use the feature evaluation method (Laplacian Score and Relief-F Score) in Section 3.1.2 to score the above 25 types of time-domain features and frequency-domain features. The evaluation results are shown in Figure 12. Comprehensively considering the evaluation results of LS and RFS, this paper chooses nine features (peak, peak-to-peak, skewness, kurtosis, F3, F4, F8, F10 and F11), which have higher scores in two evaluation methods, as part of the feature sequence. These high-scoring features describe the amplitude characteristics, fluctuation characteristics and spectral density characteristics of the voltage telemetry signals.

#### 5.2.2. Complexity Feature Analysis

To verify the effectiveness of the proposed complexity feature extraction method of Huffman-multi-scale entropy, this paper analyzes the sample entropy under different sample lengths and different scales.

**Figure 13:**
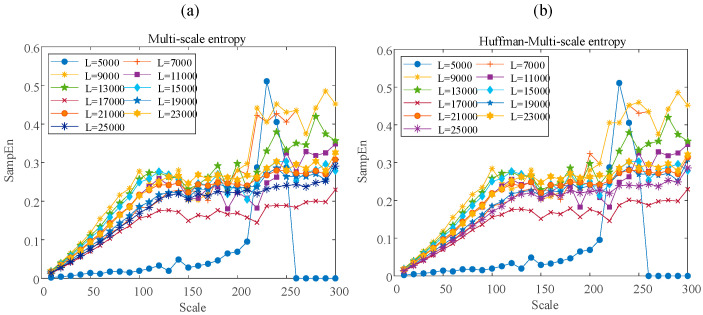
The results of the multi-scale entropy (**a**) and Huffman-multi-scale entropy (**b**) with different sample length.

Taking a normal type data Stable Change (large) as an example to study the impact of sample length on complexity characteristics, the sample length is taken from 5000 to 25,000 at intervals of 2000. Figure 13 shows the results of calculating the multi-scale entropy and Huffman-multi-scale entropy with each sample length respectively. The scale is from 10 to 300 at intervals of 10. It can be found that when the sample length is 9000 to 15,000, both methods can achieve higher sample entropy. Therefore, this paper selects the sample length as 10,000.

**Figure 14:**
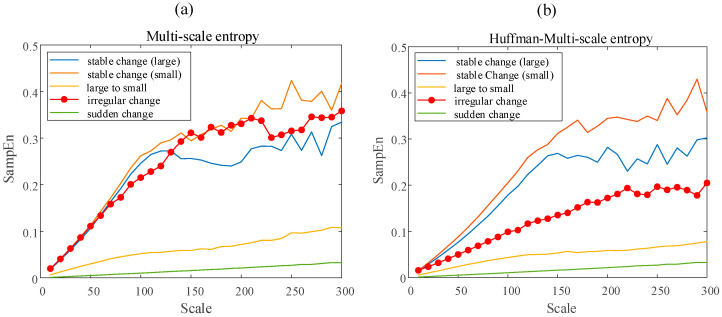
Results of the multi-scale entropy (**a**) and Huffman-multi-scale entropy (**b**) for different data type.

At the same time, this paper also calculates the multi-scale entropy and Huffman-multi-scale entropy of different types of momentum wheel voltage telemetry signals when the sample length is 10,000. The scale ranges from 10 to 300, with an interval of 10. The calculation result is shown in Figure 14. It can be seen from Figure 14 that, for normal data, the results of multi-scale entropy and Huffman-multi-scale entropy are close. For Irregular Change data, the value of Huffman-multi-scale entropy is significantly lower than that of multi-scale entropy. This shows that Huffman-multi-scale entropy has better distinguishing ability for data with high complexity. It is worth noting that, for the abnormal data of Sudden Change type, the data itself has pulse characteristics, which causes the fluctuation characteristics of the data before and after the sudden change to be concealed to a certain extent. Therefore, both multi-scale entropy and Huffman-multi-scale entropy can well describe the characteristics of increased complexity caused by sudden and large changes in data.

Based on the above analysis, this paper selects the sample length as 10,000. The feature sequence (HMSE + T/F) is composed of 30 complexity features (scale from 10 to 300 at intervals of 10) and nine time/frequency-domain features.

### 5.3. Anomaly Detection Results and Discussion

This paper uses a five-class SVM model based on the DAG method, and uses the proposed APSO to train the classification model. To verify the effectiveness of the proposed method on the spacecraft anomaly detection problem, this paper not only calculates the recognition accuracy (RA) of the classification model for each category, but also calculates the detection rate (DR), false alarm rate (FAR) and the missed alarm rate (MAR). The calculation method of RA, DR, FAR and MAR are shown in Equations (25)–(28).

(25) $$RA=\frac{Num(predicted=true)}{Num(true)}*100\%$$

(26) $$DR=\frac{Num(NN+FF)}{Num(true)}*100\%$$

(27) $$FAR=\frac{Num(NF)}{Num(N)}*100\%$$

(28) $$MAR=\frac{Num(FN)}{Num(F)}*100\%$$

where Num(predicted=true) is the total number of category predictions that are exactly the same as the true value, Num(true) is the total number of the test samples, Num(NN+FF) is the total number of the real normal data predicted as normal data and the real abnormal data predicted as abnormal data, Num(NF) is the total number of real normal data predicted as abnormal data, Num(N) is the total number of real normal data, Num(FN) is the total number of real abnormal data predicted as normal data, and Num(F) is the total number of real abnormal data.

**Figure 15:**
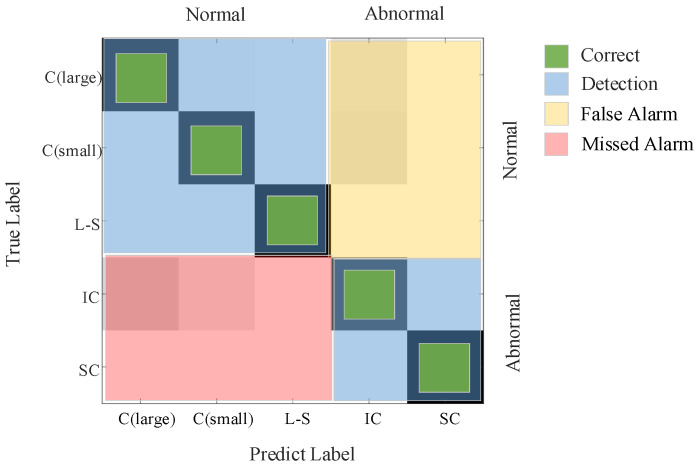
Correct, false alarms and missed alarms in the confusion matrix.

Figure 15 shows the corresponding part of the false alarm rate and the missed alarm rate in the confusion matrix. C(large) is the Stable Change (large), C(small) is the Stable Change (small), L-S is the Large to Small, IC is the Irregular Change, and SC is the Sudden Change.

**Figure 16:**
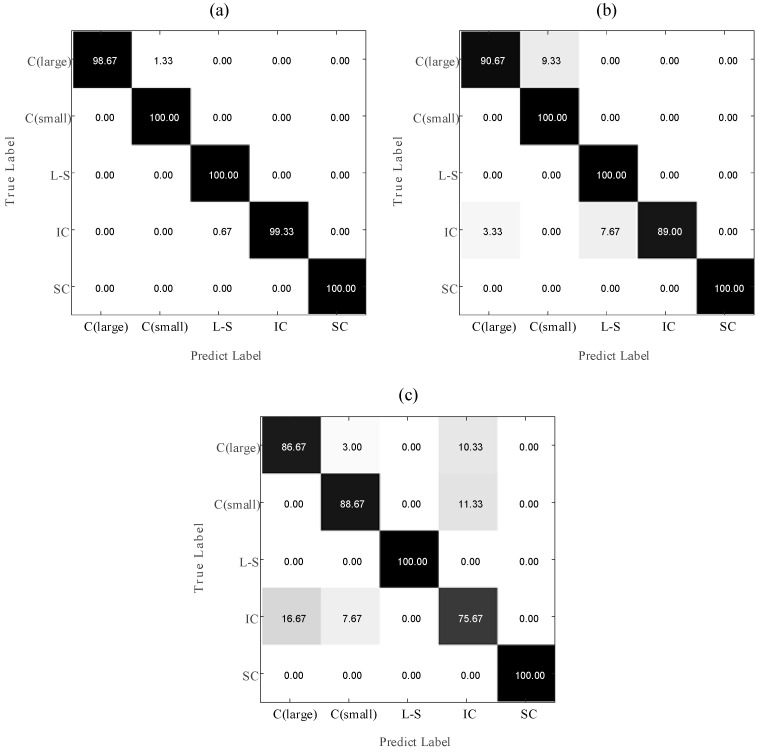
The confusion matrix of the abnormal detection for the momentum wheel voltage telemetry signal calculated by different processing methods (%): (**a**) HMSE-T/F-APSO-SVM, (**b**) HMSE-T/F-PSO-SVM, (**c**) MSE-T/F-PSO-SVM.

This paper calculates the confusion matrix of the abnormal detection of the momentum wheel voltage telemetry signal calculated by HMSE-T/F-APSO-SVM, MSE-T/F-APSO-SVM and MSE-T/F-PSO-SVM. The results are shown in Figure 16. It can be seen from the Figure 16, the identification accuracy of Sudden Change by the above three methods can all reach 100%. This result is consistent with the conclusion of the qualitative analysis of eigenvalues in the previous article. The probability of MSE-T/F-PSO-SVM identifying Stable Change (large) and Stable Change (small) as Irregular Change reaches 10.33% and 11.33%, respectively. At the same time, the probability of MSE-T/F-PSO-SVM identifying Irregular Change as Stable Change (large) and Stable Change (small) reaches 16.67% and 7.67%, respectively. The probability of HMSE-T/F-APSO-SVM and MSE-T/F-PSO-SVM identifying Stable Change (large) and Stable Change (small) as Irregular Change are 0%. At the same time, the probability of MSE-T/F-PSO-SVM identifying Irregular Change as Stable Change (large) and Stable Change (small) reaches 16.67% and 7.67%, respectively. The distinction between Stable Change (large) Stable Change (small) and Irregular Change can be effectively improved by calculating Huffman-multi-scale entropy. This conclusion is also consistent with the result in Figure 14.

In addition, the recognition accuracy of Large to Small and Sudden Change of the three methods has reached 100%, which shows that the feature sequence and anomaly detection model selected in this paper have strong sensitivity to signals with a definite change rule.

To further verify that the method proposed in this paper can effectively improve the accuracy of spacecraft anomaly detection and reduce the rate of false alarms and missed alarms, this paper compares the proposed method with other methods, and calculates the anomaly detection under different processing methods. Principal Component Analysis (PCA), Random forest (RF), Logistic Regression (LR), K-Nearest Neighbor (KNN) and Multilayer perceptron (MLP) are used in this paper. The results of the recognition accuracy, false alarm rate and missed alarm rate of different methods are shown in Table 4.

From the results in Table 4, it can be seen that: First, the recognition accuracy and detection rate of the method proposed in this paper can reach 99.60% and 99.87%, which are higher than other methods listed in the table, and the false alarm rate is reduced to 0, while the false alarm rate is reduced to 0.34%, which are lower than other methods. Second, the detection method based on the feature sequence (HMSE + T/F) has a higher recognition accuracy and detection rate as well as lower false alarm rate and missed detection rate than the detection method based on the original data. Third, by comparing the standard deviation of various indicators, it can be found that the feature sequence based on Huffman-multi-scale entropy and time-frequency domain features proposed in this paper can effectively improve the stability of the detection method.

## 6. Conclusions

In this research, we propose a new detection framework for anomaly detection based on spacecraft telemetry data. Due to the very low frequency characteristics of telemetry data, most frequency analysis methods are not suitable for spacecraft anomaly detection. Therefore, this paper first proposes a feature sequence construction method based on time-domain and frequency-domain feature screening and complexity feature fusion. On this basis, a new method of Huffman-multi-scale entropy (HMSE) based on the Huffman coding principle is proposed. To improve the classification accuracy of SVM, this paper adopts a multi-class SVM model based on the DAG principle, and proposes an improved adaptive particle swarm optimization (APSO) to train the SVM model. Then we apply the proposed method to the voltage telemetry data set of the satellite momentum wheel. Compared with other methods, the results show that the proposed method has a good performance in improving the recognition accuracy and detection rate, and it can also effectively reduce the false alarm rate and the missed alarm rate. Therefore, the method proposed in this paper has a good development prospect in the field of anomaly detection of spacecraft.

In the future work, more real-world datasets will be applied to verify the effectiveness of the detection ability of the proposed method. In addition, more methods based on artificial neural networks will be studied to further improve the versatility of anomaly detection methods.

## Figures and Tables

**Table 1:** The time/frequency-domain statistical features of x(t).

No.	Time-Domain	No.	Frequency-Domain
1	$\mathrm{peak}:\;X_{p}=\max\left\{x(n)\right\}$	14	$F_{1}=\frac{1}{K}\sum_{k=1}^{K}y\left(k\right)$
2	$\mathrm{peak}\text{-}\mathrm{to}\text{-}\mathrm{peak}:\;X_{pp}=\max\left\{x(n)\right\}-\min\left\{x(n)\right\}$	15	$F_{2}=\frac{1}{K-1}\sum_{k=1}^{K}{\left(y\left(k\right)-F_{1}\right)}^{2}$
3	$\mathrm{mean}:\;\mu =\frac{1}{N}\sum_{n=1}^{N}x(n)$	16	$F_{3}=\frac{1}{K{\left(\sqrt{F_{2}}\right)}^{3}}\sum_{k=1}^{K}{\left(y\left(k\right)-F_{1}\right)}^{3}$
4	$\mathrm{absolute}\;\mathrm{mean}:\;X_{am}=\frac{1}{N}\sum_{n=1}^{N-1}\left|x_{i}\right|$	17	$F_{4}=\frac{1}{K{\left(F_{2}\right)}^{2}}\sum_{k=1}^{K}{\left(y\left(k\right)-F_{1}\right)}^{4}$
5	$\mathrm{root}\;\mathrm{amplitude}:\;X_{ra}={\left(\frac{1}{N}\sum_{n=1}^{N}\sqrt{\left|x(n)\right|}\right)}^{2}$	18	$F_{5}=\frac{\sum_{k=1}^{K}y\left(k\right)f_{k}}{\sum_{k=1}^{K}y\left(k\right)}$
6	$\mathrm{standard}\;\mathrm{deviation}:\;\sigma =\sqrt{\frac{1}{N-1}\sum_{n=1}^{N}{\left[x(n)-\mu \right]}^{2}}$	19	$F_{6}=\sqrt{\frac{\sum_{k=1}^{K}y\left(k\right){\left(f_{k}-F_{5}\right)}^{2}}{K}}$
7	$\mathrm{root}\;\mathrm{mean}\;\mathrm{square}:\;X_{rms}=\sqrt{\frac{1}{N}\sum_{n=0}^{N}x^{2}(n)}$	20	$F_{7}=\sqrt{\frac{\sum_{k=1}^{K}f_{k}{}^{2}y\left(k\right)}{\sum_{k=1}^{K}y\left(k\right)}}$
8	$\mathrm{skewness}:\;X_{\mathrm{ske}}=\left(\frac{1}{N}\sum_{n=1}^{N}{\left(x\left(n\right)-\mu \right)}^{3}\right)/\sigma^{3}$	21	$F_{8}=\sqrt{\frac{\sum_{k=1}^{K}f_{k}{}^{4}y\left(k\right)}{\sum_{k=1}^{K}f_{k}{}^{2}y\left(k\right)}}$
9	$\mathrm{kurtosis}:\;X_{kur}=\left(\frac{1}{N}\sum_{n=1}^{N}{\left(x\left(n\right)-\mu \right)}^{4}\right)/\sigma^{4}$	22	$F_{9}=\frac{\sum_{k=1}^{K}f_{k}{}^{2}y\left(k\right)}{\sqrt{\sum_{k=1}^{K}y\left(k\right)\sum_{k=1}^{K}f_{k}{}^{4}y\left(k\right)}}$
10	$\mathrm{peak}\;\mathrm{index}:\;X_{pi}=X_{p}/X_{rms}$	23	$F_{10}=\frac{F_{6}}{F_{5}}$
11	$\mathrm{impulse}\;\mathrm{factor}:\;X_{imp}=X_{p}/X_{am}$	24	$F_{11}=\frac{\sum_{k=1}^{K}{\left(f_{k}-F_{5}\right)}^{3}y\left(k\right)}{K{\left(F_{6}\right)}^{3}}$
12	$\mathrm{margin}\;\mathrm{index}:\;X_{mi}=X_{p}/X_{ra}$	25	$F_{12}=\frac{\sum_{k=1}^{K}{\left(f_{k}-F_{5}\right)}^{4}y\left(k\right)}{K{\left(F_{6}\right)}^{4}}$
13	$\mathrm{waveform}\;\mathrm{index}:\;X_{wi}=X_{rms}/X_{am}$		

**Table 2:** An example of the Huffman coding process.

$S_{0}$	$P_{0}$	Huffman Coding	$c_{i}$	$L\left(c_{i}\right)$
$s_{1}$	0.35	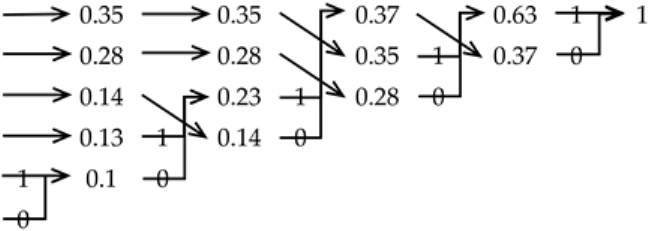	11	2
$s_{2}$	0.28		10	2
$s_{3}$	0.14		00	2
$s_{4}$	0.13		011	3
$s_{5}$	0.07		0101	4
$s_{6}$	0.03		0100	4

**Table 3:** Label description of the momentum wheel voltage telemetry dataset.

Class	Label	Health Status	Training Set	Test Set
1	Stable Change (large)	Normal	400	200
2	Stable Change (small)	Normal	400	200
3	Large to Small	Normal	400	200
4	Irregular Change	Abnormal	400	200
5	Sudden Change	Abnormal	400	200

**Table 4:** Results of the recognition accuracy, detection rate, false alarm rate and missed alarm rate of different methods (%).

Methods	Recognition Accuracy	Detection Rate	False Alarms Rate	Missed Alarms Rate
HMSE + T/F + APSO + SVM	**99.60 ± 0.28**	**99.87 ± 0.32**	**0.00 ± 0.00**	**0.34 ± 0.01**
HMSE + T/F + PSO + SVM	**95.93 ± 0.36**	**97.80 ± 0.47**	**0.00 ± 0.00**	5.50 ± 0.12
MSE + T/F + PSO + SVM	**90.20 ± 0.74**	**90.80 ± 0.39**	7.22 ± 0.85	12.17 ± 1.68
Original data + SVM	80.55 ± 2.01	83.61 ± 2.34	15.88 ± 1.58	17.16 ± 2.54
Original data + PCA	75.26 ± 4.97	77.31 ± 6.28	23.32 ± 2.69	21.74 ± 4.71
HMSE + T/F + PCA	81.75 ± 2.96	85.36 ± 3.12	12.61 ± 1.47	17.68 ± 3.63
Original data + RF	76.95 ± 4.38	80.73 ± 5.26	19.54 ± 1.85	18.86 ± 1.95
HMSE + T/F+ RF	84.28 ± 1.98	87.62 ± 2.41	14.46 ± 1.74	9.26 ± 0.36
Original data + LR	70.36 ± 4.33	72.95 ± 8.57	20.35 ± 3.54	37.1 ± 5.43
HMSE + T/F+ LR	83.78 ± 4.05	85.74 ± 4.69	10.68 ± 0.79	19.63 ± 1.37
Original data + KNN	70.37 ± 4.69	72.83 ± 5.78	24.79 ± 4.75	30.74 ± 6.35
HMSE + T/F+ KNN	83.55 ± 1.38	86.28 ± 1.54	14.82 ± 1.24	12.07 ± 1.73
Original data + MLP	86.69 ± 2.17	87.94 ± 2.24	10.67 ± 2.42	14.14 ± 1.58
HMSE + T/F + MLP	**95.25 ± 0.57**	**96.63 ± 0.68**	**0.00 ± 0.00**	8.42 ± 0.83

## Data Availability

The data included in this study are all owned by the research group and will not be transmitted.

## References

[1] Zhuang M., Tan L., Song S. (2021). Fixed-time attitude coordination control for spacecraft with external disturbance. ISA Trans..

[2] Luo Y., Cui L., Zhang J., Ma J. (2021). Vibration mechanism and improved phenomenological model of the planetary gearbox with broken ring gear fault. J. Mech. Sci. Technol..

[3] Li Z., Cheng Y., Wang H., Wang H. (2021). Fault detection approach applied to inertial navigation system/air data system integrated navigation system with time-offset. IET Radar Sonar Navig..

[4] Zhang W., Zhou J. (2019). Fault Diagnosis for Rolling Element Bearings Based on Feature Space Reconstruction and Multiscale Permutation Entropy. Entropy.

[5] Wang X., Si S., Li Y. (2021). Multiscale Diversity Entropy: A Novel Dynamical Measure for Fault Diagnosis of Rotating Machinery. IEEE Trans. Ind. Inform..

[6] Wodecki J. (2021). Time-Varying Spectral Kurtosis: Generalization of Spectral Kurtosis for Local Damage Detection in Rotating Machines under Time-Varying Operating Conditions. Sensors.

[7] Cai G., Yang C., Pan Y. (2019). EMD and GNN-AdaBoost fault diagnosis for urban rail train rolling bearings. Discret. Contin. Dyn. Syst. S.

[8] Neupane D., Seok J. (2020). Bearing Fault Detection and Diagnosis Using Case Western Reserve University Dataset with Deep Learning Approaches: A Review. IEEE Access.

[9] Hu Q., Zhang X., Niu G. (2019). Observer-based fault tolerant control and experimental verification for rigid spacecraft. Aerosp. Sci. Technol..

[10] Hou S., Sun H., Li Q., Tang X. (2021). Design and experimental validation of a disturbing force application unit for simulating spacecraft separation. Aerosp. Sci. Technol..

[11] Song B.-P., Zhou R.-D., Yang X., Zhang S., Yang N., Fang J.-Y., Song F.-L., Zhang G.-J. (2021). Surface electrostatic discharge of charged typical space materials induced by strong electromagnetic interference. J. Phys. D Appl. Phys..

[12] Boone N.R., Bettinger R.A. (2021). Spacecraft survivability in the natural debris environment near the stable Earth-Moon Lagrange points. Adv. Space Res..

[13] McGarry J.F., Carabajal C.C., Saba J.L., Reese A.R., Holland S.T., Palm S.P., Swinski J.A., Golder J.E., Liiva P.M. (2021). ICESat-2/ATLAS Onboard Flight Science Receiver Algorithms: Purpose, Process, and Performance. Earth Space Sci..

[14] Kumar R.R., Cirrincione G., Cirrincione M., Tortella A., Andriollo M. (2020). Induction Machine Fault Detection and Classification Using Non-Parametric, Statistical-Frequency Features and Shallow Neural Networks. IEEE Trans. Energy Convers..

[15] Tao L., Yang X., Zhou Y., Yang L. (2021). A Novel Transformers Fault Diagnosis Method Based on Probabilistic Neural Network and Bio-Inspired Optimizer. Sensors.

[16] Lin Y., Ge H., Chen S., Pecht M. (2020). Two-level fault diagnosis RBF networks for auto-transformer rectifier units using multi-source features. J. Power Electron..

[17] Wang T., Wang J., Wu Y., Sheng X. (2020). A fault diagnosis model based on weighted extension neural network for turbo-generator sets on small samples with noise. Chin. J. Aeronaut..

[18] Dong H., Chen F., Wang Z., Jia L., Qin Y., Man J. (2021). An Adaptive Multisensor Fault Diagnosis Method for High-Speed Train Traction Converters. IEEE Trans. Power Electron..

[19] Belagoune S., Bali N., Bakdi A., Baadji B., Atif K. (2021). Deep learning through LSTM classification and regression for transmission line fault detection, diagnosis and location in large-scale multi-machine power systems. Measurement.

[20] Oh S., Han S., Jeong J. (2021). Multi-Scale Convolutional Recurrent Neural Network for Bearing Fault Detection in Noisy Manufacturing Environments. Appl. Sci..

[21] Lv X., Wang H., Zhang X., Liu Y., Jiang D., Wei B. (2021). An evolutional SVM method based on incremental algorithm and simulated indicator diagrams for fault diagnosis in sucker rod pumping systems. J. Pet. Sci. Eng..

[22] Shi Q., Zhang H. (2021). Fault Diagnosis of an Autonomous Vehicle with an Improved SVM Algorithm Subject to Unbalanced Datasets. IEEE Trans. Ind. Electron..

[23] Han T., Zhang L., Yin Z., Tan A.C. (2021). Rolling bearing fault diagnosis with combined convolutional neural networks and support vector machine. Measurement.

[24] Lu Y., Li Y. (2021). A novel data-driven method for maintenance prioritization of circuit breakers based on the ranking SVM. Int. J. Electr. Power Energy Syst..

[25] Liu Q., Liu W., Mei J., Si G., Xia T., Quan J. (2021). A New Support Vector Regression Model for Equipment Health Diagnosis with Small Sample Data Missing and Its Application. Shock. Vib..

[26] Cuong-Le T., Nghia-Nguyen T., Khatir S., Trong-Nguyen P., Mirjalili S., Nguyen K.D. (2021). An efficient approach for damage identification based on improved machine learning using PSO-SVM. Eng. Comput..

[27] Wang B., Zhang X., Xing S., Suna C., Chena X. (2021). Sparse representation theory for support vector machine kernel function selection and its application in high-speed bearing fault diagnosis. ISA Trans..

[28] Zheng H., Wang R., Yang Y., Li Y., Xu M. (2020). Intelligent fault identification based on multisource domain generalization towards actual diagnosis scenario. IEEE Trans. Ind. Electron..

[29] Yan X., Liu Y., Ding P., Jia M. (2020). Fault Diagnosis of Rolling-Element Bearing Using Multiscale Pattern Gradient Spectrum Entropy Coupled with Laplacian Score. Complexity.

[30] Song Y., Si W., Dai F., Yang G. (2020). Weighted ReliefF with threshold constraints of feature selection for imbalanced data classification. Concurr. Comput. Pract. Exp..

[31] Richman J., Lake D., Moorman J. (2004). Sample entropy. Methods Enzymol..

[32] Costa M., Goldberger A.L., Peng C.-K. (2002). Multiscale Entropy Analysis of Complex Physiologic Time Series. Phys. Rev. Lett..

[33] Huffman D. (1952). A Method for the construction of minimum-redundancy codes. Proc. IRE.

[34] Kennedy J., Eberhart R. Particle swarm optimization. Proceedings of the ICNN’95—International Conference on Neural Networks.

